# Enhancing Security in ZigBee Wireless Sensor Networks: A New Approach and Mutual Authentication Scheme for D2D Communication

**DOI:** 10.3390/s23125703

**Published:** 2023-06-19

**Authors:** Alaa Allakany, Abeer Saber, Samih M. Mostafa, Maazen Alsabaan, Mohamed I. Ibrahem, Haitham Elwahsh

**Affiliations:** 1 Computer Science Department, Faculty of Computers and Information, Kafrelsheikh University, Kafrelsheikh 33516, Egypt; 2 Information Technology Department, Faculty of Computers and Artificial Intelligence, Damietta University, Damietta 34519, Egypt; 3 Computer Science Department, Faculty of Computers and Information, South Valley University, Qena 83523, Egypt; samih_montser@sci.svu.edu.eg; 4 Faculty of Industry and Energy Technology, New Assiut Technological University (N.A.T.U.), New Assiut City 71684, Egypt; 5 Department of Computer Engineering, College of Computer and Information Sciences, King Saud University, P.O. Box 51178, Riyadh 11543, Saudi Arabia; 6 Department of Cyber Security Engineering, George Mason University, Fairfax, VA 22030, USA; 7 Department of Electrical Engineering, Faculty of Engineering at Shoubra, Benha University, Cairo 11672, Egypt

**Keywords:** cryptography, advanced encryption standard (AES), ZigBee protocol, internet of things (IoT)

## Abstract

The latest version of ZigBee offers improvements in various aspects, including its low power consumption, flexibility, and cost-effective deployment. However, the challenges persist, as the upgraded protocol continues to suffer from a wide range of security weaknesses. Constrained wireless sensor network devices cannot use standard security protocols such as asymmetric cryptography mechanisms, which are resource-intensive and unsuitable for wireless sensor networks. ZigBee uses the Advanced Encryption Standard (AES), which is the best recommended symmetric key block cipher for securing data of sensitive networks and applications. However, AES is expected to be vulnerable to some attacks in the near future. Moreover, symmetric cryptosystems have key management and authentication issues. To address these concerns in wireless sensor networks, particularly in ZigBee communications, in this paper, we propose a mutual authentication scheme that can dynamically update the secret key value of device-to-trust center (D2TC) and device-to-device (D2D) communications. In addition, the suggested solution improves the cryptographic strength of ZigBee communications by improving the encryption process of a regular AES without the need for asymmetric cryptography. To achieve that, we use a secure one-way hash function operation when D2TC and D2D mutually authenticate each other, along with bitwise exclusive OR operations to enhance cryptography. Once authentication is accomplished, the ZigBee-based participants can mutually agree upon a shared session key and exchange a secure value. This secure value is then integrated with the sensed data from the devices and utilized as input for regular AES encryption. By adopting this technique, the encrypted data gains robust protection against potential cryptanalysis attacks. Finally, a comparative analysis is conducted to illustrate how the proposed scheme effectively maintains efficiency in comparison to eight competitive schemes. This analysis evaluates the scheme’s performance across various factors, including security features, communication, and computational cost.

## 1. Introduction

Wireless sensor networks are recognized as a vital component of IoT, representing an emerging technology with wide-ranging applications across various fields. With their implementation advancing rapidly, the importance of ensuring security becomes increasingly paramount. IoT has moved beyond its early stages and is actively reshaping our perception of the Internet from a static entity into a fully integrated and dynamic network of the future. ZigBee, Z-Wave, 6LoWPAN, and LoRa are among the communication technologies employed within the realm of IoT. The IoT industry has witnessed a significant increase in adoption, especially in ZigBee communication, which has attracted the attention of research communities to examine the security challenges faced by IoT [1]. ZigBee technology is a wireless sensor network based on the IEEE 802.15.4 standard that provides low-rate, low-power, and low-cost connectivity for various applications, including industrial automation, intelligent control, and medical health. Despite its numerous advantages, ZigBee technology encounters various challenges, including restrictions on device computing, limited memory space, and constrained energy consumption. These limitations make it impractical to use traditional security mechanisms such as asymmetric cryptography. Hence, there is a need to conduct further research in this area and increase research efforts to develop alternative security solutions.

The cryptanalytic strength of a cipher against mathematical and algebraic attacks is closely related to the length of the key material. Selecting the appropriate key length is a delicate process that involves balancing security and performance requirements. Various recommendations are available for different cryptographic ciphers and operations [2,3,4]. The National Institute of Standards and Technology (NIST) has published a report stating that all versions of the AES are expected to remain secure beyond 2030 [5,6]. In a previous report by the National Security Agency (NSA) in the IETF, symmetric ciphers with a key size of 256 bits or more were projected to remain secure until 2245 [7]. Therefore, the current version of AES requires alternative solutions to strengthen its security.

The researchers proposed several hybrid encryption techniques, which incorporate encryption schemes of two symmetric keys or both symmetric and asymmetric encryption methods. These techniques offer superior security compared with single encryption models that use public or private keys. Although many techniques that combine cryptographic algorithms are available on the market and claim to enhance data security, they may not be suitable for the constraints of wireless sensor networks.

In addition to enhancing the encryption process of ZigBee, it is essential to implement authentication and authorization measures to secure the network infrastructure. Using one-way authentication cannot guarantee security for both parties in communication. Instead, mutual authentication, where both parties are authenticated before transmission, is an effective solution to this issue. Deploying and managing robust authentication mechanisms in IoT infrastructures is considerably more challenging because of restrictions on wireless sensor devices. The literature has presented various authentication schemes to create a secure and reliable communication infrastructure for ZigBee wireless sensor networks, such as [8,9,10]. Although these mechanisms have resolved the security and privacy issues of resource-constrained networks such as ZigBee, some of these methods come with relatively higher costs of communication and computation. Additionally, several methods utilize the real identity of devices for communication, potentially establishing connections between previous and future transactions under the same identity. This compromises privacy and makes authentication more complex.

The motivation of this research lies in addressing the security weaknesses of ZigBee, improving cryptographic strength, and ensuring secure communication without the drawbacks of resource-intensive asymmetric cryptography and key management/authentication issues. The symmetric key block cipher AES currently used in ZigBee may become vulnerable in the future. Moreover, symmetric cryptosystems pose challenges related to key management and authentication. Most existing approaches involve hybrid schemes, combining symmetric and asymmetric cryptography, or using symmetric cryptography with additional costs. The proposed scheme is symmetric cryptography-based and introduces the use of secure one-way hash functions and bitwise exclusive OR operations during mutual authentication and encryption. This enables D2D via a TC to establish a shared session key and exchange a secure value, which is integrated with sensed data for regular AES encryption. The objective is to provide robust protection against cryptanalysis attacks, offer a mutual authentication solution with the unlinkability and untraceability of ZigBee devices’ communication, and maintain efficiency compared with other schemes.

To this end, in this paper, we propose a method for enhancing AES-based cryptography to address the aforementioned security challenges. Additionally, we present a mutual authentication and privacy scheme that enables each device in the ZigBee network to establish secure D2TC and D2D communication sessions. Our suggested approach achieves diverse security properties, such as anonymity and traceability, alongside the critical dual attributes of confidentiality and integrity.

To summarize our contribution:

1. Key distribution in the original version of ZigBee has serious weaknesses since keys are transmitted over the air or preinstalled onto devices in an insecure manner. Additionally, all nodes share the same key, which puts the entire network at risk if a single node is compromised. Our solution addresses these shortcomings by utilizing a one-time-use session key and a secure adynamic array of bits to secure communication between two nodes, ensuring they cannot be used for future communications.
2. We introduce a novel mutual authentication approach for ZigBee wireless sensor networks.
3. This paper proposes a solution to strengthen the encryption between D2TC and D2D communication in ZigBee wireless sensor networks.
4. The proposed approach achieves protection against various attacks by relying on simple operations rather than computationally expensive cryptographic operations.

The paper is structured as follows: Section 2 presents a brief literature review concerning schemes related to the proposed solution. Then, in Section 3 we provide a breakdown of the fundamental components necessary for understanding the security framework of ZigBee and the network model. Section 4 provides a detailed description of the proposed mutual authentication scheme and encryption process, including its phases and working mechanism, followed by an informal security analysis in the subsequent section. Section 5 presents a detailed analysis of the results obtained by comparing the proposed mutual authentication scheme with existing schemes. Finally, the paper concludes with some closing remarks.

## 2. Related Works

The ZigBee protocol used in IoT networks presents significant security and privacy challenges. To address these issues, various authentication and cryptography mechanisms have been proposed in the literature, aiming to resolve these challenges without requiring changes to the current technological infrastructure. The following paragraphs present a concise overview of relevant approaches that closely align with the proposed scheme, without delving into the specific details of these schemes.

In study research, Lee et al. [8] presented an IoT-specific lightweight mutual authentication protocol to enhance security. Instead of employing complex encryption schemes such as asymmetric encryption, the authors utilized symmetric encryption. As a result, this scheme is suitable for use with constrained IoT devices. While the paper offers a method for authenticating RFID tags with readers, it neglects the aspect of cryptography strength. Kulkarni et al. [9] suggested a secure routing protocol for ZigBee networks, wherein aggregated MAC is utilized for authentication code. It offers authentication at every step of the path, along the entire route. While it allows for end-to-end authentication, the use of only two keys fails to guarantee communication secrecy. In addition, the paper does not provide an in-depth or comprehensive analysis of the security issues in the ZigBee protocol. It focuses solely on the MAC aspect, without considering other potential vulnerabilities or attack vectors.

An IoT mutual authentication scheme designed to enhance security in IoT systems was introduced by Zhao et al. [10]. Through the implementation of dynamic password generation and the establishment of mutual authentication between devices and gateways, the scheme seeks to enhance the authentication process. The scheme underwent rigorous evaluation through mathematical analysis and simulation, demonstrating superior performance in terms of both security and efficiency compared with existing schemes. Nonetheless, it is worth noting that this scheme has several disadvantages as it fails to accommodate key properties such as anonymity, unlinkability, and untraceability. These shortcomings can undermine the privacy and security of the system, potentially exposing devices to risks such as identity disclosure, correlation of activities, and traceable transaction history. Chu et al. [11] presented a scheme leveraging a smart card and a hash function to establish secure authentication and communication between IoT devices and the network. The scheme introduces a novel key-updating mechanism that enhances system security by regularly refreshing the authentication keys. Within this proposed framework, the generation of public and private key pairs relies on ECC. The initialization phase involved the configuration and computation of elliptic curve public parameters, which were specifically designed to be utilized during the authentication phase. Nevertheless, this scheme included limited safeguards for the aforementioned critical security properties.

Gaikwad et al. [12] focused on implementing a Kerberos-based authentication scheme with three levels of security for an IoT smart home system. The authors proposed the utilization of symmetric algorithms, precisely the hash algorithms and AES, to elevate the level of security within the system. However, it is important to note that the work did not encompass D2D interactions. The absence of secure communication and authentication among network devices poses a restriction, considering their vital role in ensuring the system’s overall security. Additionally, this approach does not adequately address important security properties such as transaction anonymity, lack of linkability, and absence of traceability. These properties are crucial for maintaining privacy and preventing the identification and tracking of users’ activities within the smart home system. Ashibani et al. [13] introduced authentication frameworks for smart homes that incorporate context awareness. In their dynamic authentication scheme, the security of local and remote access to IoT smart home devices is enhanced by integrating traditional static credentials and a range of contextual information. However, their work primarily focuses on device-to-cloud interactions and lacks authentication for device-to-device interactions.

Mishra et al. [14] presented a resilient authentication protocol that employs smart cards within a wireless sensor network based on the internet of things. In the proposed protocol, the establishment of authentication between the node acting as the gateway and the node serving as the sensor is accomplished through the utilization of password hash values and pre-shared keys. The authors emphasized the successful achievement of user anonymity and demonstrated the protocol’s resilience against various attacks. However, it is worth noting that this work does not address authentication in D2D interactions. Alshahrani and Traore [15] developed a mutual authentication and automated access control scheme specifically for IoT smart homes, focusing on lightweight implementation, incorporating a cumulative keyed hash. The scheme employed the controller node for authenticating the nodes, enabling the establishment of a temporary session key in a manner that ensures anonymity and prevents linkability. The authors leveraged fog computing architecture, which was modified to prevent identity theft attacks. This adaptation of fog architecture played a crucial role in ensuring secure and trustworthy IoT device identities. However, the protocol did not take into account instances where the IoT node leaves a specific home network and joins a different network. It was stated that this concern will be addressed in future research, aligning with our own objectives.

Chang and Le [16] introduced an authentication scheme tailored for ad hoc wireless sensor networks, focusing on achieving both security and efficiency. Their scheme provides provable security guarantees while maintaining an efficient authentication process. However, the scheme cannot guarantee the property of untraceability. This limitation arises from the fixed parameter in the login message, which remains the same for different sessions. Consequently, an adversary can readily deduce that both messages originate from the same user. Exploiting this vulnerability, the adversary can trace and monitor the user’s activities, compromising their privacy and anonymity.

Alalak et al. [17] presented a solution to remove the vulnerabilities present in block cipher key encryption algorithms, incorporating support for multiple keys. This allows the system to encrypt each data block with a distinct key, ensuring no two blocks are encrypted using the same key. By implementing this method, the occurrence of two plaintexts being encrypted with the same cipher key is eliminated, thus reducing the opportunities for analysts to exploit the ciphertext. However, it is important to note that this approach requires additional storage space.

A three-factor authentication protocol for IoT environments was proposed by Mirsaraei et al. [18]. The protocol incorporates blockchain technology, hashing functions, XOR operations, and the fuzzy extractor concept. By leveraging these cryptographic techniques, the protocol achieves a suitable level of security, safeguards data from tampering, and enhances the transparency of recorded information on smart cards. Through their research, the authors demonstrated the effectiveness of the proposed protocol in ensuring secure mutual authentication. To perform formal analysis, the authors employed BAN logic, the ROR model, and the Avispa tool. In future research, we also intend to employ these three tools for the formal analysis of our own protocol.

The low-power nature of sensor nodes and the intermittent wireless connections between them create vulnerabilities that can be exploited by low-rate denial of service (LDoS) attacks, causing nodes to become unavailable. In their work [19], Gong et al. introduced a mutual identity authentication scheme for the IoT to defend against such attacks. Similar to our approach, the protocol proposed in their paper not only defends against common attacks but also ensures secure traceability while maintaining anonymity.

In their study, Amor et al. [20] presented a novel approach aimed at creating a secure social industrial IoT (SIIoT) system. Their proposed system enables mutual authentication among social IoT devices. While our approach shares commonalities with theirs, utilizing symmetric cryptography, hash functions, and bitwise XOR operations, it is important to highlight that the strength of the employed cryptographic algorithm was not taken into account in their approach.

### Discussion

Several research studies have explored the potential of utilizing lightweight cryptographic functions, such as hash functions and bitwise XOR, for various purposes. However, these studies have overlooked the need for strong mutual authentication with minimal computational and communication cost, which is crucial for ZigBee devices. Consequently, the development of an efficient mutual authentication framework remains a significant challenge within the IoT ecosystem. Furthermore, it is important to note that a significant portion of these approaches prioritize strong mutual authentication without addressing the improvement of encryption or considering vital properties such as anonymity, linkability, and traceability. In contrast, our proposed approach not only considers a solution for D2TC and D2D authentication in ZigBee protocol, it also provides a solution to enhance encryption. Additionally, our approach effectively tackles crucial security properties, including transaction anonymity, absence of linkability, and lack of traceability.

## 3. ZigBee Network Architecture

Within this section, we present a summary of both the ZigBee security architecture and the network model utilized in this paper.

### 3.1. Security Architecture

ZigBee is an economical wireless sensor network with a low power-consumption design, consisting of four layers. The initial two layers, the physical (PHY) layer and the media access control (MAC) layer, are responsible for the fundamental functions of the physical radio and facilitate communication between two devices within a single-hop connection. These layers comply with the IEEE 802.15.4 standard. Additionally, the network (NWK) layer is incorporated to handle tasks such as packet routing and address management. Finally, at the topmost layer, the application (APL) layer defines the node’s role within the network and ensures the establishment and maintenance of secure links between nodes [21,22,23].

ZigBee PRO and ZigBee represent two feature sets available within the ZigBee framework. ZigBee PRO is designed to support larger networks with over a thousand devices while maintaining low power consumption, making it a widely accepted specification. One notable and innovative aspect of ZigBee PRO is its inclusion of green power, enabling the seamless integration of energy harvesting devices that operate without the need for external power supplies [24].

Within the ZigBee protocol, three key types serve security purposes: (1) The network key, also known as the NWK key, is a master key that is shared among all devices within a ZigBee network. It enables network-wide encryption and decryption capabilities. The NWK key is established and distributed to all devices during the network initialization phase. (2) The link key is an exclusive key shared between two devices in a ZigBee network, ensuring secure communication specifically between those devices. Derived from the NWK key, link keys are frequently used for encryption and decryption purposes in pairwise communication. (3) The master key is a high-level key in a ZigBee network, playing a crucial role in secure provisioning and key establishment. During the initial setup phase, the master key guarantees the secure distribution of the network key, facilitating the establishment of trust between devices. These key types have critical roles in securing communication, providing authentication, and safeguarding data privacy within ZigBee networks.

To ensure system confidentiality, ZigBee technology implements the AES 128-bit algorithm within the MAC layer. AES is a symmetric block cipher algorithm used for encrypting data. While numerous applications currently utilize the AES algorithm to secure their systems against data disclosure, NIST researchers have predicted the potential vulnerability of the AES 128-bit encryption algorithm by 2036 due to rapid advancements in computing technology [7,17]. ZigBee technology incorporates the enhanced CCM* mode to ensure both confidentiality and integrity. This advanced version of CCM provides robust security features, including defense against replay attacks. In the following section, we outline our suggested solution to rectify the mentioned weaknesses.

### 3.2. Network Model

**Figure 1:**
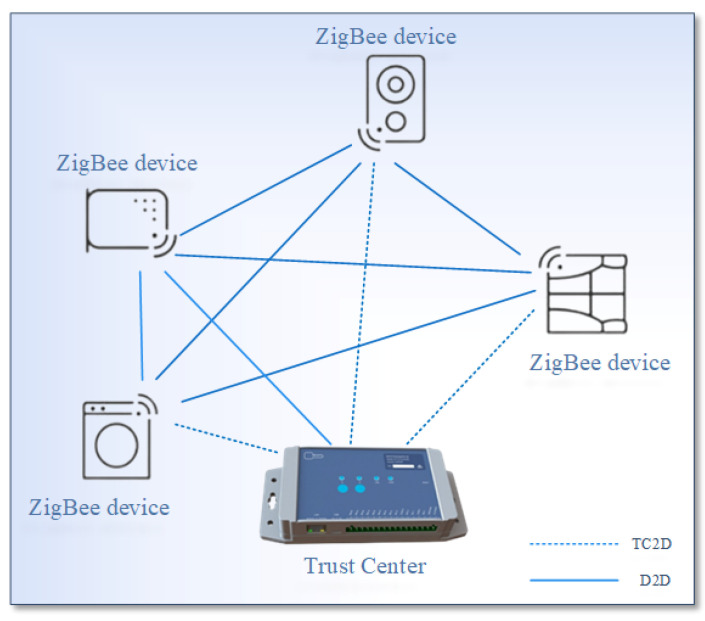
ZigBee network model.

The ZigBee network topology consists of three main types: star topology, tree topology, and mesh topology. Star or mesh topologies are commonly used as the primary architecture in ZigBee networks [25]. In mesh networks, as shown in Figure 1, TC assumes the role of both routing traffic and managing devices on the network. End devices can communicate with the network through the TC.

The TC is responsible for managing information about each device and initiating and maintaining the network, since it is aware of all devices within its network. During initialization, authentication through the TC is required for each ZigBee device, and all communication between devices must go through the TC to establish a session encryption key and for authentication purposes. Consequently, every ZigBee network must have a TC. ZigBee devices typically interact with the physical world or with other devices present in the network. Our approach employs the method presented in Section 4.2 and Section 4.3 for authentication of both TC2D and D2D. Additionally, encryption takes place using the AES algorithm with a key length of 128 bits, as demonstrated in Section 4.4.

## 4. Proposed Solution

Our proposed solution consists of two primary stages. The first stage is the device authentication and key agreement phase, which includes three sub-phases: (i) offline phase, (ii) authentication phase, and (iii) communication phase. The second stage involves strengthening the cryptography based on AES by utilizing a secure value exchanged in the first stage to enhance the cryptography of exchanged data in the ZigBee network. We provide a detailed description of both stages below, and we use the notations presented in Table 1 to describe our solution throughout the paper.

### 4.1. Offline Phase

To establish secure D2TC and D2D communication in ZigBee networks, every device (Di) is required to register its master key and identity ID with the relevant TC during an offline phase to prevent unauthorized devices from gaining access. Only devices that have been registered in the offline phase are permitted to initiate a communication session.

### 4.2. Device Authentication Phase

**Figure 2:**
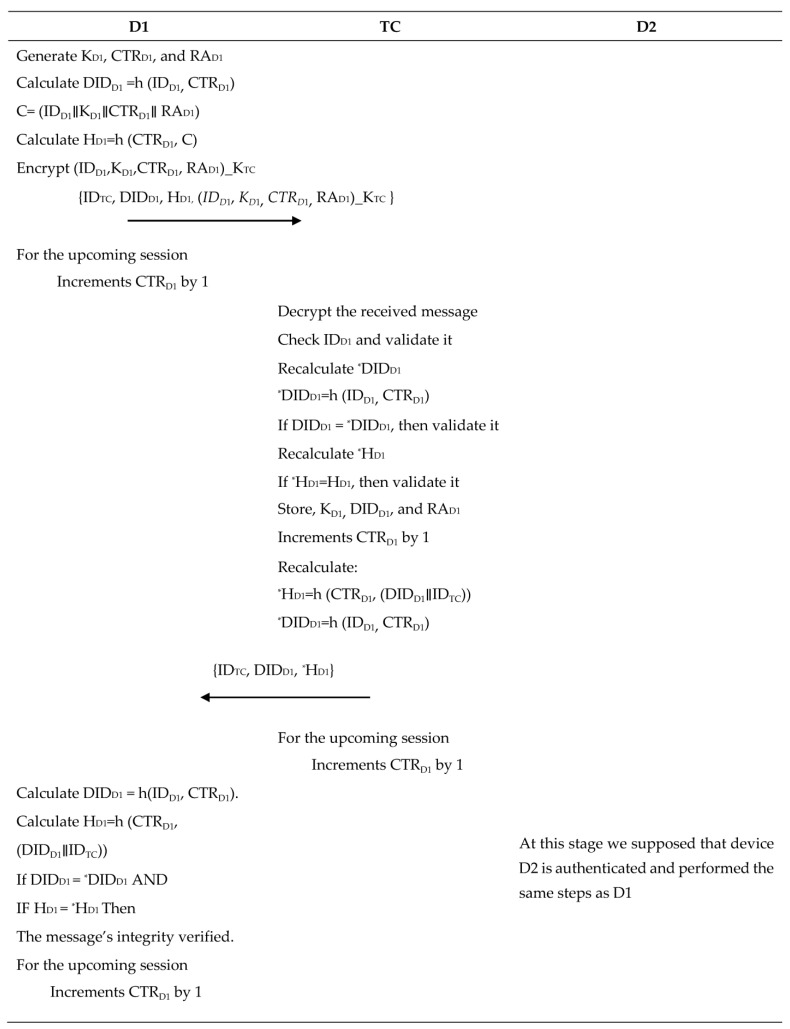
A detailed description of D1 and D2 registration and authentication phase.

In this section, D2TC authentication process is described. During the offline phase, if a device (Di) has participated, its ID address should be available in the authenticated devices list that is stored in the TC’s memory. If the ID matches one from the stored list, the requesting device (Di) will receive an encrypted message to begin the communication process. In our proposed solution, we prioritize the security and protection of ZigBee devices’ real identities (IDs) by ensuring that they are never transmitted in plain text. This is achieved by exclusively utilizing dynamic IDs during both the authentication and communication phases. The steps in this phase are explained below and summarized in Figure 2.

Step1: Device to trust center

The device D1 generates three parameters, namely, K_D1_, CTR_D1_, and RA_D1_. K_D1_ is the key that will be used for encrypting the exchanged data with D1. The counter CTR_D1_ is incremented with each message. RA_D1_ is a random array of bytes equal to the block size used in AES. The device D1 creates a dynamic identity DID_D1_ = h(ID_D1_, CTR_D1_) that changes for every session. This method ensures that the DID_D1_ is unique in every session and can only be traced by the TC. Then D1 concatenates ID_D1_, K_D1_, CTR_D1_, and RA_D1_ and calculates the hash value, H_D1_ = h(CTR_D1_, (ID_D1_∥K_D1_∥CTR_D1_∥RA_D1_)). This ensures that H_D1_ is computed based on two dynamic values, enabling the detection of attacks even if one of the values is compromised. Finally, D1 encrypts (ID_D1_, K_D1_, CTR_D1_, RA_D1_) using the K_TC_ and sends the message {ID_TC_, DID_D1_, H_D1,_ (ID_D1_, K_D1_, CTR_D1_, RA_D1_)_K_TC_ } to the TC. 

Step 2: Trust center to device

After receiving the message, the TC decrypts it using K_TC_, looks up ID_D1_, and subsequently carries out a validation process. Then, TC calculates the hash value of ^*^H_D1_ and DID_D1_ and proceeds to compare them against the received H_D1_ and DID_D1_ for verification. The TC stores D1 information, K_D1_, CTR_D1_, DID_D1_, and RA_D1_ in its database. Then, the TC increments CTR_D1_ by 1. Next, it concatenates the (DID_D1_∥ID_TC_). After that, the TC computes the hash value, H_TC1_ = h(CTR_D1_, (DID_D1_∥ID_TC_)). Then, the TC sends the message to D1. Finally, the TC increments CTR_D1_ again by 1 and computes for the upcoming session DID_D1_ = h(ID_D1_, CTR_D1_). On the other side, D1 computes both the hash value and dynamic identity of D1. Then, it verifies whether these values match those received from the TC. When a match is identified, it signifies the accurate validation of the message’s integrity. Finally, D1 updates the CTR_D1_ value and the new dynamic identity for the upcoming session. At the end of this stage, as explained in steps 1 and 2, both D1 and the TC authenticate each other, and the parameters (ID_D1_, CTR_D1_, DID_D1_, and RA_D1_) are securely sent between D1 and TC. The second stage describes the communication among devices. We assume that the ZigBee device D1 wants to communicate as a sender with the ZigBee device D2 as a receiver.

### 4.3. D2D Communication Phase

In this section, we discuss the D2D communication phase, during which a device verifies the identity of one or more devices. Once verified, the devices establish a shared key and a secure array of bits that can be employed later to establish an encrypted communication channel. The following steps discuss the communication process in this phase.

Step 1: Device (D1) to device (D2)

The device D1 sends a request to establish communication with an authenticated device D2. The message contains the device’s dynamic identity DID_D1_, the identity of trust center ID_TC_, and the identity of the second device ID_D2_ encrypted using K_D1_. Then, the D1 computes the hash value of those parameters and CTR_D1_, H_D1_ = h (CTR_D1_, (ID_TC_∥DID_D1_∥(ID_D2_)_k_TC_). 

Step 2: Device (D2) to trust center

After constructing its message using the same method, device D2 forwards both the information it received from D1 and its own information to the trust center, requesting a temporary session key.

Step 3: Trust center authentication

The messages received by TC from D2 are validated by reconstructing H_D1_ and H_D2_ with K_D1_, K_D2_, CTR_D1_, and CTR_D2_ for the corresponding stored DID_D1_ and DID_D2_. The comparison of the hash value computed at TC, H_TC_ for both D1 and D2, and matching with H_D1_ and H_D2_ confirms the message is authentic, as only D1 and D2 have access to K_D1_, K_D2_, CTR_D1,_ and CTR_D2_. In addition, they have the ability to construct a valid message. 

Step 4: Trust center to device (1) and trust center to device (D2)

TC creates the session key KS_D12_ and random array of bits RA_D12_, and subsequently sends them to D1 and D2 in an encrypted form using K_D1_ and K_D2,_ respectively. 

Step 5: Devices (D1 and D2) authentication

After receiving the encrypted information, D1 and D2 retrieve the secret session key using their respective private keys K_D1_ and K_D2_. The presence of CTR_D1_ and CTR_D2_ in the received message assures both devices that the message is new. At this stage, both devices can communicate securely utilizing the session key KS_D12_. Figure 2 provides a summary of the steps outlined in the preceding explanation.

**Figure 3:**
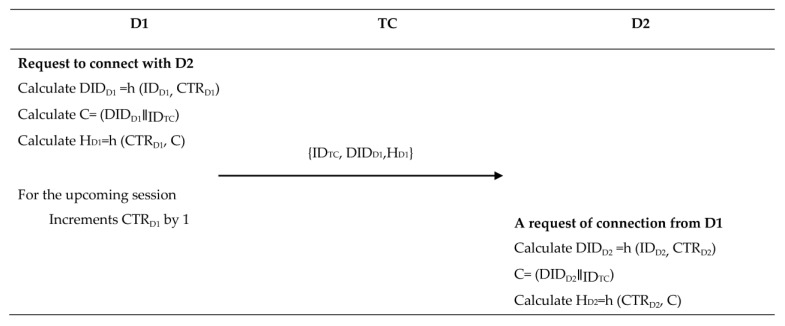

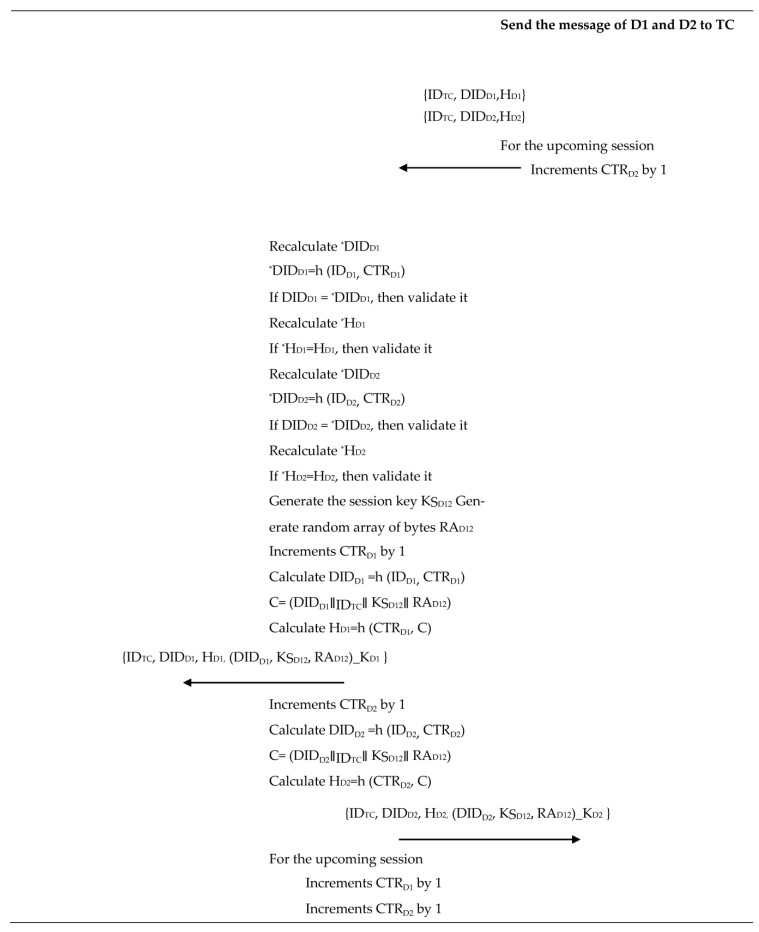
A detailed description of D1 and D2 registration phase.

Remark: We assume that the generated random array of bytes that will be used to strength the cryptography of AES will be used in the encryption process at the point that the devices need to send sensed data to the TC or other devices in the ZigBee network. The steps of this phase are explained below and are summarized in Figure 3.

### 4.4. Encryption Based on AES

**Figure 4:**
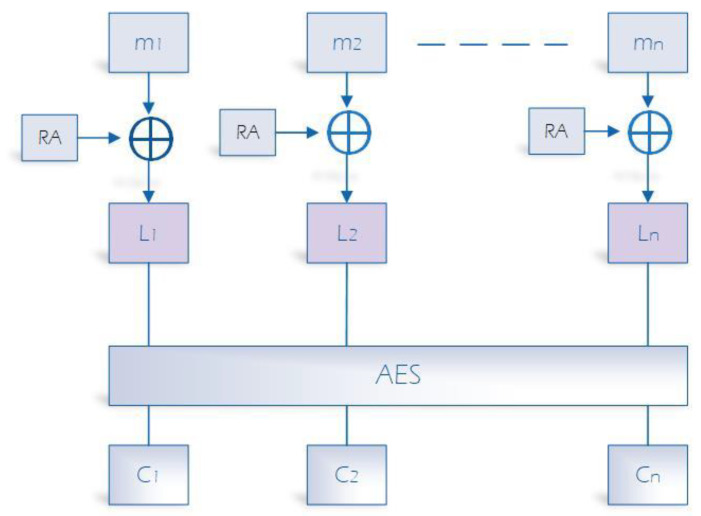
Encryption in proposed solution based on AES.

The main idea involves producing a random array of bits that matches the block size used in AES. This array will be combined with newly produced data by the ZigBee device, and the resulting output will serve as input for a standard AES. By doing so, the array will be refreshed with each message transmission between D2TC or D2D in the ZigBee network, ensuring that it remains unique for each session and can only be accessed by the two parties involved in the communication. The updated array, i.e., the previous array merged with the data generated by the ZigBee device, will be stored in both the device and the TC to be utilized in the encryption process of the subsequent message exchange between the two parties. This technique makes it difficult to perform cryptanalysis on the encrypted messages. The randomly generated array, which is updated with each sensed data, can offer protection against cryptanalysis attacks, even if the symmetric key is breached. The following figure summarizes our encryption solution (Figure 4).

In our solution, the sensed data of the ZigBee device will be divided into blocks of size 128 bits. Then, the random generated array will be merged with each block and the output of this step will be used as input to the regular AES 128/CCM algorithm. Since the data are encrypted, the device will send the message to the receiver device in the ZigBee network and update the array as follows: RA = Ln. On the other side, the receiver device will decrypt the message based on the pre-shared symmetric key and RA, then recalculate RA and store it in its database for the upcoming session. In the following section, we explain how the session symmetric key and RA are exchanged and updated, in addition to the process of device authentication and communication.

## 5. Security and Performance Analysis

In these subsections, we validate the security of our proposed approach through informal security analysis and static performance assessment.

### 5.1. Informal Security Analysis

This section provides a comprehensive analysis of potential attacks and their prevention strategies in the proposed approach.

#### 5.1.1. Access Control

Maintaining control and access restrictions is crucial to prevent potential attacks resulting from unauthorized network access. Our proposed solution involves the trust center authenticating devices that intend to join the network. During initialization, the trust center permits access solely to the device that possesses the master key and the pre-stored device identity. Subsequently, the trust center sends its key (K_TC_) to the device, encrypted with the master key, and initiates the authentication process to generate a new device key.

#### 5.1.2. Eavesdropping Attack

Messages exchanged between the trust center and the device during the authentication phase are susceptible to interception by attackers. However, our approach addresses this vulnerability by employing symmetric encryption to encrypt the message, which we have strengthened further by modifying the standard AES algorithm. Additionally, we leverage dynamic identities that change with each session, making it challenging for the attacker to establish a connection between the message and a particular device.

#### 5.1.3. Replay Attacks

To prevent replay attacks, our solution employs different random numbers in each session during message exchanges. An eavesdropper’s attempts to impersonate the trust center and replay previous responses are prevented by this approach. Our approach effectively mitigates replay attacks as the device rejects messages containing a non-fresh nonce that does not match the nonce in the request message.

#### 5.1.4. Anonymity and Un-Traceability

To prevent the attacker from linking an identity to a particular device and tracing specific messages to a given device, our solution utilizes a unique random number selected by the device in each session to compute the dynamic identity of the device. Furthermore, we ensure that sensitive data, such as the real identity of a device, are always encrypted. As a result, using random values such as CTR and RA for computing dynamic identity and hash functions ensures the preservation of anonymity for all messages transmitted via public channels by the device or the trust center. Moreover, if the same device sends two or more authentication messages, the ability of the attacker to determine whether these messages originate from the same device is eliminated. Consequently, there is no possibility of linking the device to different sessions, which enhances the anonymity of our proposed approach and thwarts the attacker’s ability to trace devices through message interception.

Table 2 summarizes the security attacks addressed by our proposed approach. Additionally, a comparative analysis of the proposed approach and existing approaches based on various security features is presented in Table 3.

### 5.2. Static Performance Assessment

This section focuses on analyzing the efficiency and overhead of the proposed approach with regards to its communication overhead and storage requirements. Our approach not only offers numerous security features to protect against potential attacks on wireless sensor networks, namely the ZigBee network, but also incurs a reasonable computational cost, making it suitable for communication and storage-constrained wireless sensor networks.

#### 5.2.1. Computation Cost

In Table 4, we provide a summary of the computation cost analysis, where we compare our proposed approach to other related solutions based on the computational cost of hash and XOR operations. From Table 4, it can clearly be seen that our approach required 20 hash operations, which is less than some solutions. Furthermore, unlike all other solutions, our approach does not involve any XOR operations. Our analysis demonstrates that our proposed approach is computationally efficient, making it suitable for wireless sensor devices with limited resources, such as ZigBee devices.

#### 5.2.2. Communication Cost

Along with computational costs, communication costs are also a crucial consideration in authentication schemes for wireless sensor networks, as they determine the bandwidth necessary for transmitting packets during authentication. Our approach employs SHA-1 as the hash function with an output size of 160 bits. Additionally, DID_D_, CTR, RA_D_, K_D_, and ID_D_ are set to 160, 160, 128, 128, and 16 bits, respectively, while ID_TC_ is 16 bits long.

When transmitting (D → TC), D sends a tuple consisting of {ID_TC_, DID_D1_, H_D1,_ (ID_D__1_, K_D__1_, CTR_D__1_, RA_D1_)_K_TC_}. The size of this transmitted tuple is calculated as ((3 × 160) +(2 × 16) + (2 × 128)) = 768 bits. In contrast, when transmitting (TC → D), TC sends a tuple of {ID_TC_, DID_D1_, ^*^H_D1_} with a size of ((2 × 16) + 160) = 192 bits. In the case of transmitting (D1→ D2), D1 sends a tuple of {ID_TC_, DID_D1_, H_D1_} with a size of (16 + (2 × 160)) = 336 bits. Following that, (D2→ TC) of the same size = 336 bit. Finally, during transmission (TC→ D1), TC sends a tuple of {ID_TC_, DID_D1_, H_D1,_ (DID_D1_, KS_D12_, RA_D12_)_K_D1_} with a size of (16 + (3 × 160) + (2 × 128)) = 752 bits, which is also the same size for the transmission (TC→ D2) = 752 bits. As a result, the total communication cost is 3136 bits.

The communication cost analysis presented in Table 5 demonstrates that the proposed approach has a lower cost compared with other approaches [26,27,28,29,30], and a higher cost than approach [18]. However, our approach incorporates an additional parameter, a secret array of 128 bits, to enhance the cryptography, which is not utilized in [16] and provides advantages to our approach.

## 6. Conclusions

In this paper, we propose a mutual authentication and key agreement approach for ZigBee wireless sensor networks. ZigBee devices are known for their low-power, low-cost, and lightweight characteristics. To optimize energy efficiency, we have exclusively employed hash functions for mutual authentication between D2TC and D2D, along with bitwise exclusive OR operations to enhance cryptography. Moreover, our approach uses the dynamic identity of the devices to preserve anonymity, while providing confidentiality, integrity, and untraceability properties. Through an informal security analysis, we have verified the resilience of our approach against commonly encountered attacks. Additionally, our approach’s efficiency was evaluated through a comparative analysis, comparing it with other relevant solutions.

In our future work, we aim to extend the protocol to handle cases where a ZigBee device switches from one network to another. Furthermore, we intend to investigate scenarios where multiple ZigBee TCs exist within the network. We aim to experimentally investigate and evaluate the performance overhead for those cases and conduct a formal security analysis using BAN logic, the ROR model, and the AVISPA tool [18].

## Figures and Tables

**Table 1:** Description of Notations.

Notations	Description
D1	End device 1
D2	End device 2
ID_Di_	Real identity of device Di
DID_Di_	Dynamic identity of device Di
*DID_Di_	Dynamic identity of device Di calculated at TC
RA_Di_	Random array of bytes generated at device Di
CTR_Di_	Counter value generated at device Di
TC	Trust center
K_Di_	Device Di symmetric key
K_TC_	Trust center’s key
KS_D12_	The session key between device D1 and D2
H_D1_	Hash value calculated at device D1
*H_Di_	Hash value calculated at TC sent to Di
∥	Concatenation
C	Several concatenated parameters
⊕	Bitwise XOR

**Table 2:** Security attack analysis.

Attack	Description
Mutual authentication	Mutual authentication is required for all parties involved in communication in the data exchange phase to establish trust.
Device anonymity	Throughout the communication process, the real identity of the device, i.e., its ID, is never transmitted. Instead, a dynamic identity (DID) is generated for the communication process.
Eavesdropping attack and untraceability	Our scheme includes encryption of information at each transmission stage, making it impossible for an attacker to access the original content even if they intercept an ongoing transmission. Furthermore, we enhance the encryption process by adding a secret value.
Message integrity	By utilizing hashed values, we can detect message modification as any alteration to the original message content will result in a change in the hash values.
Replay attack	To prevent replay attacks, our scheme uses a different session key in addition to a dynamic secret value in every transmission, ensuring that identical messages cannot be transmitted in future sessions.

**Table 3:** Analysis of the security parameters.

Attack	[16]	[26]	[27]	[28]	[29]	[30]	Proposed Approach
Device anonymity and untraceability	No	No	No	Yes	Yes	Yes	Yes
Replay attack	Yes	No	Yes	Yes	Yes	Yes	Yes
Forward secrecy	No	Yes	Yes	Yes	No	Yes	Yes
Message integrity attack	Yes	Yes	Yes	Yes	Yes	Yes	Yes
Strength of cryptography	No	No	No	No	No	No	Yes

**Table 4:** Comparison of the computation cost.

Phases	[16]	[26]	[27]	[28]	[29]	[30]	[31]	Proposed Approach
Sensor/D1	5Ch + 4C_XOR_	5Ch + 3C_XOR_	3Ch + 1C_XOR_	3Ch + 7C_XOR_	6Ch + 1C_XOR_	4Ch + 4C_XOR_	7C_h_	6C_h_
User/D2	7Ch + 4C_XOR_	12Ch + 7C_XOR_	14Ch + 7C_XOR_	--	7Ch + 1C_XOR_	7Ch + 4C_XOR_	10C_h_	2C_h_
Server/Trust center	8Ch + 1C_XOR_	15Ch + 7C_XOR_	9Ch + 4C_XOR_	1Ch + 12C_XOR_	10Ch + 2C_XOR_	5Th + 3C_XOR_	17C_h_	12C_h_
Total cost	20Ch + 9C_XOR_	32Ch + 17C_XOR_	26Ch + 12C_XOR_	7Ch + 19C_XOR_	23Ch + 4C_XOR_	16Ch + 11C_XOR_	34C_h_	20C_h_

Sensor/D1: in some approaches it represents the device (D1) side but in others approaches it represents the sensor side. User/D2: represent the user side or the device (D2) side. Server/Trust center: represents the server or trust center side. Ch: Computational cost of hash operations. CXOR: Computational cost of XOR operations.

**Table 5:** Comparison of the communication cost.

Approach	Communication Cost
[16]	3104 bits
[26]	4096 bits
[27]	3184 bits
[28]	4672 bits
[29]	4800 bits
[30]	3808 bits
[31]	3040 bits
Proposed	3136 bits

## Data Availability

The data that support the findings of this study are available on request from the corresponding author.

## References

[1] Orfanos V.A., Kaminaris S.D., Papageorgas P., Piromalis D., Kandris D. (2023). A Comprehensive Review of IoT Networking Technologies for Smart Home Automation Applications. J. Sens. Actuator Netw..

[2] (2006).

[3] Traore M. (2022). Analyse des biais de RNG pour les mécanismes cryptographiques et applications industrielles. Cryptographie et Sécurité [cs.CR].

[4] Cryptographic Key Length Recommendations. http://www.keylength.com.

[5] (2001). Advanced Encryption Standard (AES).

[6] (2006). Recommendation for KeyManagement.

[7] (2004). Recommendation for the Triple DataEncryption Algorithm (TDEA) Block Cipher.

[8] Lee J.Y., Lin W.C., Huang Y.H. A lightweight authentication protocol for internet of things. Proceedings of the 2014 International Symposium on Next-Generation Electronics (ISNE).

[9] Kulkarni S., Ghosh U., Pasupuleti H. Considering security for ZigBee protocol using message authentication code. Proceedings of the 2015 Annual IEEE India Conference (INDICON).

[10] Zhao G., Wang X., Si J., Long X., Hu T. A novel mutual authentication scheme for internet of things. Proceedings of the 2011 International Conference on Modelling, Identification and Control (ICMIC).

[11] Chu F., Zhang R., Ni R., Dai W. An improved identity authentication scheme for internet of things in heterogeneous networking environments. Proceedings of the 2013 Sixteenth International Conference on Network-Based Information Systems.

[12] Gaikwad P.P., Gabhane J.P., Golait S.S. 3-level secure Kerberos authentication for smart home systems using IoT. Proceedings of the 2015 First International Conference on Next Generation Computing Technologies (NGCT).

[13] Ashibani Y., Kauling D., Mahmoud Q.H. A context-aware authentication framework for smart homes. Proceedings of the 2017 IEEE Thirtieth Canadian Conference on Electrical and Computer Engineering (CCECE).

[14] Mishra D., Vijayakumar P., Sureshkumar V., Amin R., Islam S.H., Gope P. (2018). Efficient authentication protocol for secure multimedia communications in IoT-enabled wireless sensor networks. Multimed. Tools Appl..

[15] Alshahrani M., Traore I. (2019). Secure mutual authentication and automated access control for IoT smart home using cumulative Keyed-hash chain. J. Inf. Secur. Appl..

[16] Chang C.-C., Le H.-D. (2015). A Provably Secure, Efficient, and Flexible Authentication Scheme for Ad hoc Wireless Sensor Networks. IEEE Trans. Wirel. Commun..

[17] Alalak S., Ahmed Z., Abdullah A., Subramiam S. (2011). Aes and ecc mixed for zigBee wireless sensor security. Int. J. Electron. Commun. Eng..

[18] Mirsaraei A.G., Barati A., Barati H. (2022). Asecure three factorauthentication scheme for IoT environments. J. Parallel Distrib. Comput..

[19] Gong B., Zheng G., Waqas M., Tu S., Chen S. (2023). LCDMA: Lightweight Cross-domain Mutual Identity Authentication Scheme for Internet of Things. IEEE Internet Things J..

[20] Amor A.B., Jebri S., Abid M., Meddeb A. (2022). A secure lightweight mutual authentication scheme in social industrial IoT environment. J. Supercomput..

[21] Yang B. Study on security of wireless sensor network based on ZigBee standard. Proceedings of the International Conference on Computational Intelligence and Security.

[22] Qianqian M., Kejin B. Security analysis for wireless networks based on ZigBee. Proceedings of the 2009 International Forum on Information Technology and Applications.

[23] Misic J., Misic V. (2008). Wireless Personal Area Networks: Performance, Interconnections and Security with IEEE, 2008, 802.15.4.

[24] Varghese J.M., Rao N., Varghese V.T. (2015). A survey of the state of the art in ZigBee. Int. J. Cybern. Inform..

[25] Haque K.F., Abdelgawad A., Yelamarthi K. (2022). Comprehensive Performance Analysis of ZigBee Communication: An Experimental Approach with XBee S2C Module. Sensors.

[26] Amin R., Islam S.H., Biswas G.P., Khan M.K., Leng L., Kumar N. (2016). Design of an anonymity-preserving three factor authenticated key exchange protocol for wireless sensor networks. Comput. Netw..

[27] Gope P., Hwang T. (2016). A realistic lightweight anonymous authen-tication protocol for securing real-time application data access in wireless sensor networks. IEEE Trans. Ind. Electron..

[28] Li X., Ibrahim M.H., Kumari S., Sangaiah A.K., Gupta V., Choo K.K.R. (2017). Anonymous mutual authentication and key agree- ment scheme for wearable sensors in wireless body area net-works. Comput. Netw..

[29] Wu F., Li X., Xu L., Kumari S., Karuppiah M., Shen J. (2017). A lightweight and privacy-preserving mutual authentication scheme for wearable devices assisted by cloud server. Comput. Electr. Eng..

[30] Ankur G., Meenakshi T., Jamil S.T., Aakar S. (2019). A lightweight anonymous user authentication and key establishment scheme for wearable devices. Comput Netw..

[31] Fotouhi M., Bayat M., Das A.K., Far H.A.N., Pournaghi S.M., Doostari M. (2020). A lightweight and secure two-factor authentication scheme for wireless body area networks in health-care IoT. Comput. Netw..

